# Supplementary material for: A machine learning approach in a monocentric cohort for predicting primary refractory disease in Diffuse Large B-cell lymphoma patients
Source: PLoS One. 2024 Oct 1;19(10):e0311261. doi: 10.1371/journal.pone.0311261 (PMC11444388; doi:10.1371/journal.pone.0311261)
Supplement: Attachment — Submitted filename: REBUTAL LETTER Machine LEarning DLBCL.docx [file pone.0311261.s002.docx]

Dr Marie DETRAIT

Department of Technology and Informations Systems, Grand Hopital de Charleroi

June 28, 2024

To Editorial Board of PlosOne

Dear Editor,

Subject : Response to Reviewers’Comments on Manuscript ID [**PONE-D-24-07639**]

We sincerely thank you for the valuable feedback you provided on our manuscript titled « [**A Machine learning approach in a monocentric cohort for predicting primary refractory disease in Diffuse Large B-Cell Lymphoma patients**] ». We have carefully considered each comment and have made the necessary revisions to address them. Below, we provide detailed responses to each reviewers’s comments and yours.

**• Provide a comprehensive explanation and reporting of model performance measures (calibration and discrimination) in both training and validation datasets for all five ML models to allow for a thorough critical evaluation.**

In this study, the models were evaluated using the validation set. This study was primarily a feasability study, and there was no dataset with data unseen by the algorithms. In the ongoing prospective study, a test set containing data unseen by the algorithms has been reserved for the purpose of final validation and potential deployment into production, provided the results are satisfactory. The article, metrics, and models have been revised in light of the recommendations (TRIPOD-AI). We have expanded the discussion and included a comprehensive report detailing the strengths and weaknesses of the models (Table 4) to better reflect their perfomance.

Otherwise, we have set up a repository on Github with the dataset and the informatics code at [**https://github.com/MarieDetrait-MD/PrimaryRefractoryDLBCL**](https://github.com/MarieDetrait-MD/PrimaryRefractoryDLBCL)**.**

**• Expand the discussion to consider existing evidence, study strengths and limitations, and potential barriers to further implementation in clinical settings.**

The discussion has been expanded to address these questions and requests.

**• Organize the manuscript coherently and conduct a thorough grammar and style review to correct typos in the manuscript (e.g., "septic choc" and "TEP-Scanner").**

The article has been revised accordingly.

**Reviewer #1**:

**1.The manuscript requires language refinement to correct grammatical errors and improve overall readability.**

The manuscript has undergone a thorough language refinement process to correct grammatical errors enhance overall readability.

**2.It would be beneficial for the authors to provide a flowchart outlining the structure of the paper to help readers gain a comprehensive overview.**

A comprehensive flowchart outlining the structure of the paper has been included to provide readers with a clear overview. This flowchart represents the key components and flow of the study.

**3.The authors should offer a more detailed discussion on the advantages and disadvantages of the five machine learning models employed in the study.**

We have provided an expanded discussion on the advantages and disadvantages of the five machine learning models employed in the study.

**4.Since the analysis includes ROC curves, it is advisable for the authors to plot these curves for clarity.**

ROC curves for the five machine learning models have been plotted and included in the manuscript. These plots are presented in the ‘Results‘ section, providing a visual representation of the models’ performance aiding in the comparison of their effectiveness.

**5.Before introducing the methodology, the authors should cite literature on machine learning for predicting DLBCL to demonstrate the feasibility of their approach.**

The literature review has been expanded and recent references have been added for machine learning research in prediction, prognosis cancer and diffuse large B cell lymphoma. The literature review demonstrates the feasability of our approach and positions our study within the context of existing research in diffuse large B cell lymphoma and machine learning. These citations are included in the ‘Introduction’ section.

References :

8.Gaur K, Jagtap MM. Role of Artificial Intelligence and Machine Learning in Prediction, Diagnosis, and Prognosis of Cancer. (2022) Cureus. 14(11):31008.

9. Zhang B, Shi H, Wang H. Machine Learning and AI in Cancer Prognosis, Prediction, and Treatment Selection: A Critical Approach. (2023) J Multidiscip Healthc. 16:1779-1791.

12.Steen CB, Luca BA, Esfahani MS, Azizi A, Sworder BJ, et al. (2021) The landscape of tumor cell states and ecosystems in diffuse large B cell lymphoma. Cancer Cell. 39(10):1422-1437.

13. Zhu Y, Ning Z, Li X, Lin Z. Machine learning algorithms identify target genes and the molecular mechanism of matrine against diffuse large B-cell lymphoma. (2024) Curr Comput Aided Drug Des. 20(6):847-859.

**6.A comprehensive discussion on the strengths and limitations of the study is warranted, including any potential areas for enhancement.**

The discussion has been reviewed to adress this point.

**7.The current literature lacks recent citations from the past three years. Therefore, it is essential to incorporate more up-to-date references to enrich the study.**

The manuscript has been updated to include recent citations from the past three years. These up-to-date references enrich the study by incorporating the latest findings and advancements in the field. These citations are included in the ‘introduction’ section.

References :

8.Gaur K, Jagtap MM. Role of Artificial Intelligence and Machine Learning in Prediction, Diagnosis, and Prognosis of Cancer. (2022) Cureus. 14(11):31008.

9. Zhang B, Shi H, Wang H. Machine Learning and AI in Cancer Prognosis, Prediction, and Treatment Selection: A Critical Approach. (2023) J Multidiscip Healthc. 16:1779-1791.

12.Steen CB, Luca BA, Esfahani MS, Azizi A, Sworder BJ, et al. (2021) The landscape of tumor cell states and ecosystems in diffuse large B cell lymphoma. Cancer Cell. 39(10):1422-1437.

13. Zhu Y, Ning Z, Li X, Lin Z. Machine learning algorithms identify target genes and the molecular mechanism of matrine against diffuse large B-cell lymphoma. (2024) Curr Comput Aided Drug Des. 20(6):847-859.

**Reviewer #2 :**

**1. It would be better if the authors include the ROC curve figures to have an illustration that allows visually compare the performance among different models.**

The Roc-AUC figure for for the five machine learning models have been plotted and included in the manuscript. These plots are presented in the ‘Results ‘ section, providing a visual representation of the models’ performance aiding in the comparison of their effectiveness.

**2. When the author describe the univariate analysis, I think it preferred to show the p-values as recommended:"There are two ways to report p values: (a) report p value based on the α level determined, e.g., “p > 0.05 or p < 0.05” or “p > 0.01 or p < 0.01” and (b) report the exact p value (the posteriori probability reported by the statistical software). If the exact p value is less than 0.001, it is conventional to state merely p < 0.001"**

The p value has been corrected.

**3. What is the F-score described in the univariate analysis ? is it the same as F1-Score? it is not clear to me, as it is described in integers for the different features included in the model and not the F1-Score for the model itself. Please clarify**

The F-score was Feature-importance. The F1-score for each model has been added and discussed. A reference has been added to characterize the different measures.

24 Sokolova M, Lapalme G (2009) A systematic analysis of performance measures for classification tasks. Inf. Process. Manage. 45(4):427-437.

**4. F1-score should be included in table 3.**

F1-score has been included.

**5. I understand that you share the code only in specific cases, trying to preserve confidentiality for patient data. However, I believe that if you want your model to be validate by other groups, a website/blog with a tool where a clinician might introduce data an have a prediction using your model would be ideal. For doing that you don't have to share patient data. This is a suggestion that the authors may consider, but it is not compelled to the authors to fulfill.**

We have set up a repository on Github with the dataset and the informatics code at [**https://github.com/MarieDetrait-MD/PrimaryRefractoryDLBCL**](https://github.com/MarieDetrait-MD/PrimaryRefractoryDLBCL)**.**

In conclusion, we believe that the revisions made have significantly improved the manuscript’s quality and clarity. We appreciate the opportunity to address the reviewers’s comments and thank the editorial board for their consideration of our revised manuscript.

Please find attached the revised manuscript, which incorporates all suggested changes. We look forward to your favorable consideration of our manuscript for publication in PlosOne.

Sincerely,

Dr Marie DETRAIT

[marie.detrait@ghdc.be](mailto:marie.detrait@ghdc.be)
