# Supplementary material for: A machine learning approach in a monocentric cohort for predicting primary refractory disease in Diffuse Large B-cell lymphoma patients
Source: PLoS One. 2024 Oct 1;19(10):e0311261. doi: 10.1371/journal.pone.0311261 (PMC11444388; doi:10.1371/journal.pone.0311261)
Supplement: Attachment — Submitted filename: Response to Reviewers.docx [file pone.0311261.s003.docx]

Dr Marie DETRAIT

Department of Technology and Informations Systems, Grand Hopital de Charleroi

August 26, 2024

To Editorial Board of PlosOne

Dear Editor,

Subject: Response to Editor’s Comments on Manuscript ID [**PONE-D-24-07639**]

We would like to express our sincere gratitude for the insightful feedback provided on our manuscript titled « [**A Machine learning approach in a monocentric cohort for predicting primary refractory disease in Diffuse Large B-Cell Lymphoma patients**] ». We have carefully reviewed each comment and have made the necessary revisions to address them comprehensively.

Below, we provide detailed responses to the specific points raised:

**1. The authors have changed the model performance measures from the original submission to the first revision (R1) without providing an adequate explanation.**

We apologize for not providing a clear explanation initially. The modification was due to a change in the data preparation process. Originally, age was discretized, but when preparing the revised version, we decided to retain the original continuous age variable, as age categories were already pre-defined (age category one (18-40), age category two (40-60), age category three (60-80), age category four (>80)). This change, indeed, had an impact on the models’ performance metrics. We appreciate your attention to this detail, and we should have communicated this change in our first rebuttal letter.

**2. There is no clear comparison of ML models to existing published prognostication models beyond AUC. While Logistic regression appears to underperform, models seem to overestimate their performance without proper explicability. This is recommended per original TRIPOD statement and update TRIPOD-AI. Please see: BMJ 2024; 385 doi:** [**https://doi.org/10.1136/bmj-2023-078378**](https://doi.org/10.1136/bmj-2023-078378)

a. To enhance explainability of our models, we analyzed the most important features using the ‘feature-importance’ attribute for both the XGBoost and Random Forest models. Additionally, we examined the log-likelihoods of conditional probabilities across different classes and variables using the ‘feature_log_prob_’ attribute in the Naive Bayes classifier. This analysis helped us identify the variables that contributed most significantly to the model’s predictions. We then compared these variables with established prognostic indicators in the literature. We found that the variables utilized by the Naive Bayes classifier were particularly relevant for accurate predictions. Moreover, the IPI score and age were consistently identified as significant across all three models. These findings have now been integrated into the manuscript and are discussed in detail.

b. We recognize that our comparison of models in the current study primarily focused on AUC and other common performance metrics. A comprehensive comparison with existing prognostic models from the literature, beyond these metrics, was not fully explored. Although the Naive Bayes classifier and other models demonstrated promising results based on standard metrics, we acknowledge the importance of ensuring explainability and transparency of model behavior, particularly in clinical contexts. Moving forward, including in our ongoing prospective study, we plan to conduct a more thorough comparison with established prognostic models, particularly focusing on the IPI score, cell of origin, and biomarkers such as BCL2, MYC, and BCL6. We also intend to enhance the interpretability of our models. Specifically, we will employ the LIME (Local Interpretable Model-agnostic Explanations) technique, which will be implemented in Python for our prospective study, to ensure that model decisions are transparent and can be endorsed by clinicians.

It is important to note that this study was primarily a feasibility study and did not involve a dataset containing data unseen by the algorithms. However, in the ongoing prospective study, we have set aside a test set with unseen data for final validation to ensure the absence of overfitting.

We appreciate the reference to the “TRIPOD+AI statement : updated guidance for reporting clinical prediction models that use regression or machine learning methods“  by Gary S collins et al., which provides invaluable guidance for our ongoing and future studies. Thank you for highlighting this crucial aspect, which we agree warrants discussion in our manuscript.

We have also added a reference for the LIME technique as follows:

33. Ribeiro MT, Singh S, Guestrin C (2016) “Why should I trust you?”: explaining the predictions of any classifier. Proceedings of the 22^nd^ ACM SIGKDD International Conference on Knowledge Discovery and Data Mining. New York: ACM, 1135-1144.

**3. An additional author has been added to the original list without a proper explanation.**

You are correct in noting this discrepancy. Dr. V. Robin was inadvertently omitted when the authors’ names were initially submitted online; although she was correctly listed as an author in the manuscript itself. This oversight has been corrected, and we appreciate your diligence in bringing this to our attention.

In conclusion, we believe that the revisions made have significantly improved the quality of our manuscript. We appreciate the opportunity to address these important comments and thank you and the editorial board for your thoughtful consideration of our revised manuscript. Please find attached the revised manuscript, which incorporates all suggested changes. We look forward to your favorable consideration of our manuscript for publication in PlosOne.

Sincerely,

Dr Marie DETRAIT

[marie.detrait@ghdc.be](mailto:marie.detrait@ghdc.be)
